# New software and breast boundary landmarks to calculate breast volumes from 3D surface images

**DOI:** 10.1007/s00238-018-1431-2

**Published:** 2018-07-06

**Authors:** T. S. Wesselius, R. D. Vreeken, A. C. Verhulst, T. Xi, T. J. J. Maal, D. J. O. Ulrich

**Affiliations:** 1 Department of Plastic-, Reconstructive-, and Hand Surgery, Radboud University Medical Center, P.O. Box 9101, 6500 HB Nijmegen, the Netherlands; 2 Radboudumc 3D Lab, Radboud University Medical Center, Nijmegen, the Netherlands; 3 Department of Oral and Maxillofacial Surgery, Radboud University Medical Center, Nijmegen, the Netherlands

**Keywords:** 3D stereophotogrammetry, 3D surface imaging, 3D analysis, Breast volume, Breast landmarks

## Abstract

**Background:**

A method to accurately calculate breast volumes helps achieving a better breast surgery outcome. 3D surface imaging potentially allows these calculations in a harmless, quick, and practicable way. The calculated volume from a 3D surface image is dependent on the determined breast boundary and the method of chest wall simulation by software. Currently, there is no consensus on a robust set of breast boundary landmarks and validation studies on breast volume calculation software are scarce. The purposes of this study were to determine the robustness of newly introduced breast boundary landmarks and introduce and validate a new method to simulate a chest wall.

**Methods:**

Sixteen subjects who underwent a unilateral simple mastectomy were included. In addition to the natural skin fold of the breast, the sternomanubrial joint, the transition of the pectoral muscle curve into the breast curvature, and the midaxillary line were used as landmarks to indicate the breast boundary. The intra- and interrater variability of these landmarks was tested. Furthermore, new chest wall simulation software was validated on the breastless chest side of the subjects.

**Results:**

The intra- and interrater variability of the three breast boundary markers was small (mean 3.5–6.7 mm), and no significant difference was found between the intra- and interrater variability (*p* = 0.08, *p* = 0.06, and *p* = 0.10). The mean volume error of the most accurately simulated chest wall was 4.6 ± 37 ml.

**Conclusion:**

The newly introduced landmarks showed to be robust and our new chest wall simulation algorithm showed accurate results.

Level of Evidence: Level IV, diagnostic study.

## Introduction

In order to achieve satisfactory results in breast surgery, such as breast reductions, augmentations, and reconstructions, a thorough understanding of the breast shape and breast volume is crucial. Many surgical decisions are based on a subjective estimation of the breast shape and volume [1]. To improve the predictability of the postsurgical result, it is highly desired to have a method to accurately calculate breast volumes preoperatively.

Previously described methods to calculate the breast volume such as water displacement [2–4], breast casts [5, 6], anthropomorphic measurements [7], mammography [8], or MRI scans [9] are often tedious, costly, time consumptive, or yield widely varying results. The evolution of 3D imaging techniques in the past decennium, 3D stereophotogrammetry in particular, provides a new approach of capturing 3D surface images. In 3D stereophotogrammetry, multiple digital cameras are used to capture an object under various angles and subsequently reconstruct a 3D surface image. Since this technique is accurate, harmless, quick, and increasingly available in clinics [10–12], it is promising for routine breast volume calculations.

Besides having a validated tool to perform the measurement of breast volume, the two other most significant factors determining the outcome are the indicated breast boundary and the simulated chest wall behind the 3D breast surface that enable the volume calculation.

**Fig. 1:**
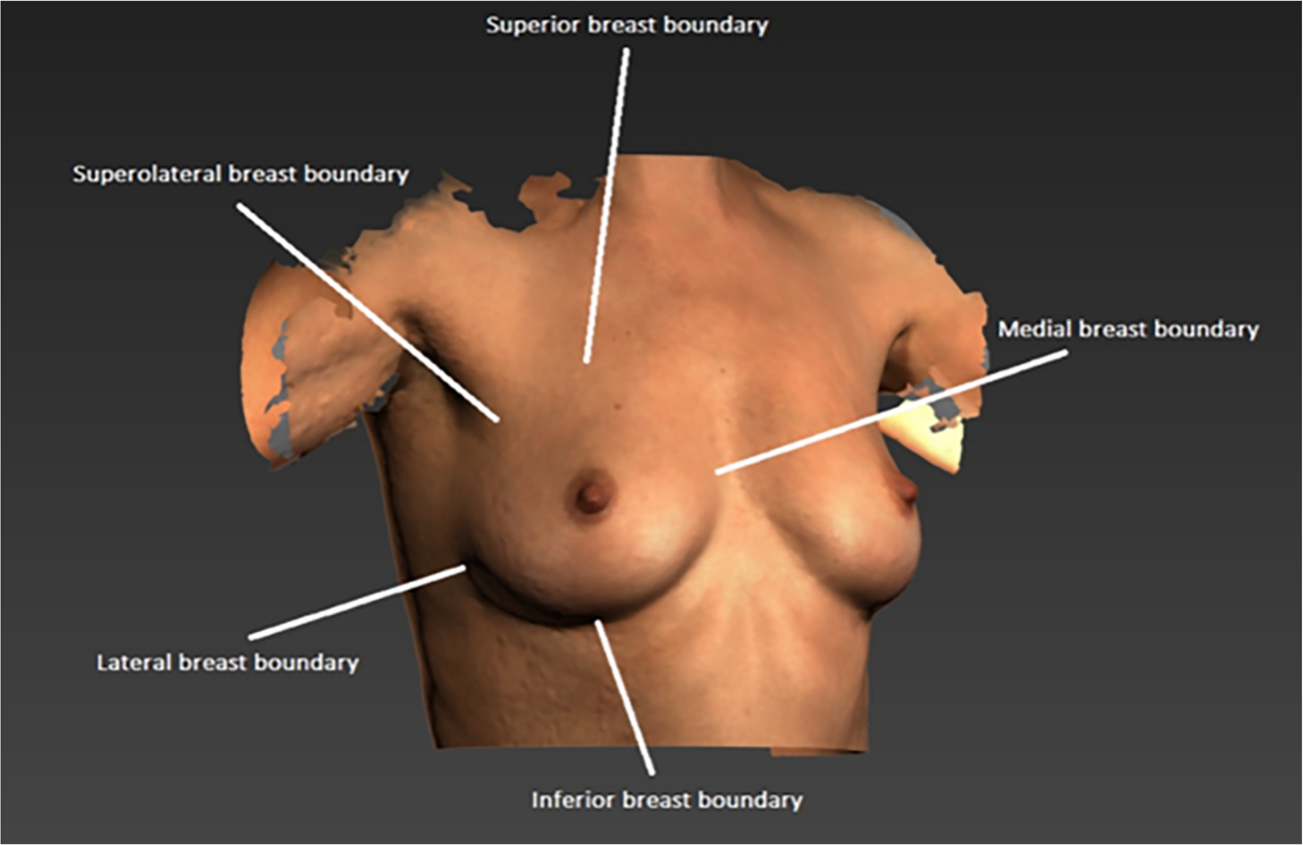
Breast boundaries. Note that the superior- and superolateral boundary are the most difficult to define

Because breast boundaries determine the size and shape of the simulated chest wall, the selected breast boundaries significantly influence the calculated breast volume from 3D surface images. Therefore, it is important to accurately determine the breast boundaries when breast volume calculations are made from 3D surface image. Because of the natural skin fold of the breast, the medial and inferior breast boundaries can easily be identified (Fig. 1). However, due to the lack of clear anatomical landmarks, the superior-, superolateral-, and lateral breast boundaries leave room for ambiguous interpretations. This has contributed to the inconsistent use of breast boundary landmarks in literature [10, 13–19].

**Fig. 2:**
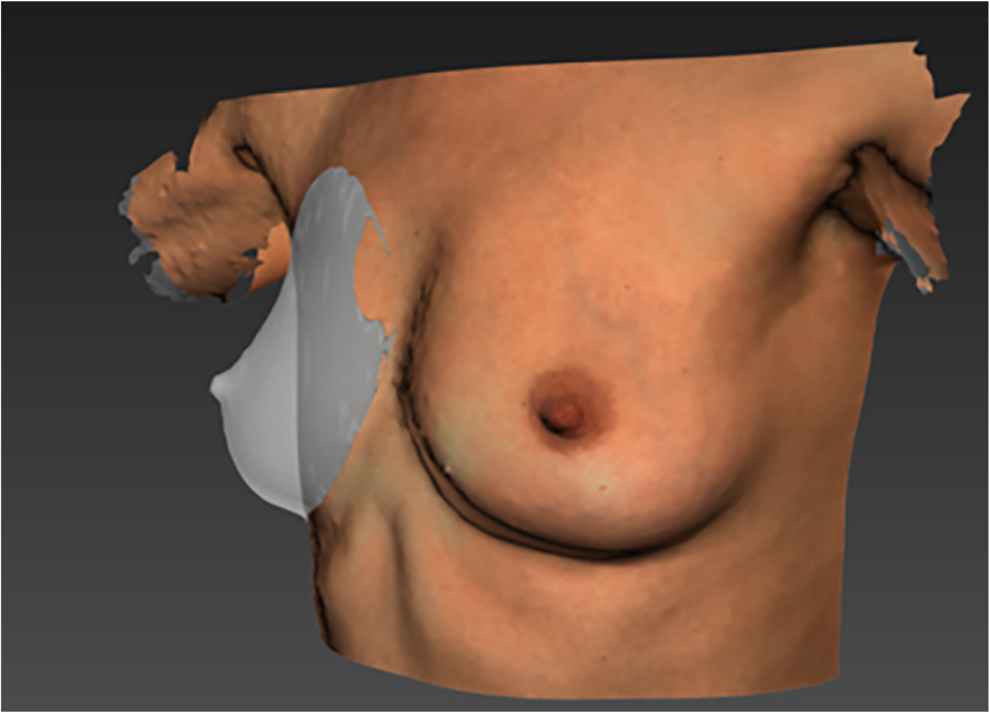
Example of a simulated chest wall behind the right breast. The breast was made transparent to make the simulated chest wall visible. The simulated chest wall determines the closed volume of the breast and is therefore essential for calculating breast volumes from a 3D surface image

In addition to the landmarks that define the boundaries of a breast, a dorsal cut-off plane that simulates the chest wall is required to make the breast volume measurement (Fig. 2). Simulating such a surface within the breast boundaries can be done with various software algorithms. Some studies used a Coons patch algorithm [12, 20], some others developed their own algorithm [21], while others used software tools without mentioning the exact algorithm used [10, 14, 19, 22, 23].

Because the algorithm for simulating a chest wall determines the calculated volume, validation and testing of the robustness of these algorithms are crucial. Chest wall algorithms have been validated by comparing the calculated breast volume with the resection weight [10, 12] or with the chest wall from an MRI [14]. However, this does not actually validate whether the simulated chest wall accurately describes the situation where skin, pectoral muscles, and rib cage are present without breast tissue. In our opinion, the gold standard for validating a simulated chest wall should be an actual breastless female chest wall.

This study introduces a set of landmarks for the identification of lateral-, superolateral-, and superior breast boundaries and is aimed at the evaluation of the intra- and interrater variability of these landmarks. Furthermore, a new algorithm to simulate a chest wall is presented, along with a new validation method for chest wall algorithms. Our custom made 3D Breast Analyzing Software Tool (3D BreAST) is based on these landmarks and the mentioned chest wall simulation algorithm.

## Materials and methods

### Subjects

Sixteen female subjects who underwent a unilateral simple mastectomy, i.e., removal of the breast tissue, nipple, and a small piece of overlying skin, and routine 3D surface imaging were included in this study (Table 1). Subjects with severe chest wall deformations due to scarring were excluded. Subjects with a high body mass index (BMI) have a substantial amount of fat that deforms the chest wall. Therefore, subjects with a BMI greater than 30 were excluded. The medical ethical committee of the Radboud University Medical Center approved this study and all subjects gave written informed consent for the usage of their data. All data were anonymized and de-identified prior to analysis.

**Table 1:** Characteristics of the included subjects (*n* = 16)

Subject #	Age (years)	BMI (kg/m^2^)	Right breast history	Left breast history
1	55	24.3	Mastectomy + RT	–
2	46	28.0	Mastectomy	–
3	57	26.3	Mastectomy	–
4	65	19.8	Mastectomy + RT	–
5	53	27.6	Mastectomy + RT	–
6	40	25.4	Mastectomy + RT	–
7	46	22.0	Mastectomy + RT	–
8	39	21.3	Mastectomy	–
9	49	23.2	Mastectomy	–
10	56	20.8	Mastectomy	–
11	48	24.3	–	Mastectomy
12	62	23.4	–	Mastectomy
13	46	23.2	–	Mastectomy + RT
14	34	28.3	Mastectomy	–
15	39	27.1	–	Mastectomy
16	44	18.8	Mastectomy	–

*BMI* body mass index, *RT* radio therapy

### Landmark locations

The axillary tail of the breast is orientated towards the midaxillary line. It is independent of the breast shape and can be located by using the middle of the armpit. The superolateral breast boundary is defined by the transition of the pectoral muscle curve into the breast curvature when the subject spreaded her arms horizontally. The upper breast tissue boundary is located at the height of the second rib. The second rib is attached to the sternomanubrial joint, which is very well palpable by its bony edge. Therefore, the upper boundary of both breasts was defined to be at the level of the bony edge of the sternomanubrial joint.

### Placing multiple sets of breast boundary landmarks

Five round stickers with a diameter of 8 mm were placed on the landmark locations described in the paragraph above. To prevent movement of the superolateral landmark due to the skin mobility in that area, the landmark was placed when the subject was in the same pose as when the 3D surface image would be captured.

To determine the intra- and interrater distance variability of the landmark placing, all landmarks were placed by two observers. On every subject, observer 1 placed the set of landmarks twice and observer 2 placed the landmarks once. Due to slight difference in pose, two 3D surface images of a subject are practically impossible to superimpose accurately. To determine the intra- and interrater variability from a single 3D surface image, all three landmark sets needed to be in place on the subject. However, this introduced a possible bias for observer 2 when he sees the landmarks placed by observer 1.

**Fig. 3:**
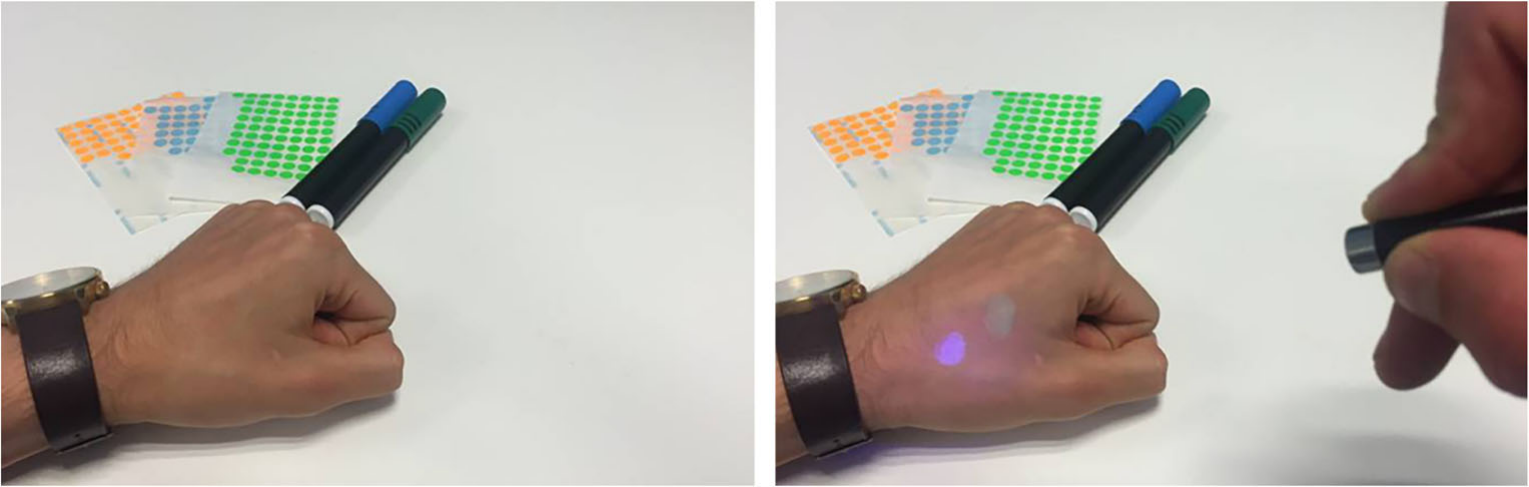
Glow in the dark skin markers. The ink of the markers is invisible in daylight, but become visible when illuminated with blacklight. These markers were used to enable the placement of multiple landmark sets on the subject without the observers seeing previously placed sets

A solution to this problem was found by using glow in the dark skin markers. The ink of glow in the dark skin markers is only visible when it is illuminated with a blacklight (Fig. 3). By using this ink for the first landmark set of observer 1 and observer 2, the bias of knowing where other marker sets were placed was dismissed. A green and blue markers were used for the first two sets. The second landmark set of observer 1 was directly marked with the 8-mm round stickers with a green color. Subsequently, the blacklight was used to detect the first landmark set of observer 1 and observer 2 to place orange and blue stickers on them, respectively.

### Acquiring 3D surface images

3D surface images of the torso were acquired with a four-pod stereophotogrammetry system consisting of 12 cameras (3dMD™, Atlanta, USA). The subjects were instructed to spread their arms horizontally and exhale normally.

### Calculating landmark reproducibility

To obtain the intra- and interrater variability for each of the five landmarks, the Euclidean distances between the marker sets were calculated with the built-in measuring lint tool from Autodesk 3ds Max Studio 2016 (Autodesk Inc., CA, USA).

Because the lateral boundary is independent from shifts in superior-inferior direction, only the ventral-dorsal direction was taken into account when calculating the distance between these landmarks. For similar reasons, only the superior-inferior direction was taken into account for the upper boundary.

### Algorithm to simulate the chest wall

**Fig. 4:**
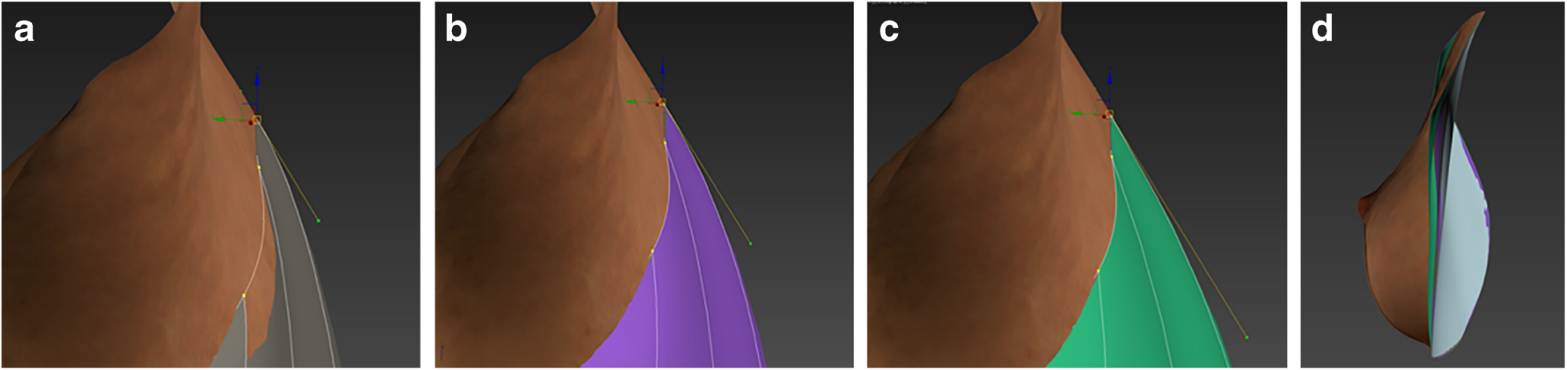
The effect of the Bézier curve handle length on the curvature of the simulated surface. The images **a**, **b**, and **c** show a right lateral view of a female torso where the breast is cut off and a chest wall is simulated with three different Bézier curve handle lengths (yellow line with green knot). The handle lengths are a fraction of the total length of the curve. **a** Bézier curve handle length = 0.20. **b** Bézier curve handle length = 0.33. **c** Bézier curve handle length = 0.50. **d** Left lateral view of the right breast with all three surfaces in place, showing the differences between them

The algorithm to simulate a chest wall behind a breast was written in Autodesk 3ds Max’s Maxscript. The algorithm creates a surface that is based on a framework of Bézier curves that run from the upper breast boundary to the lower breast boundary. The handle lengths of each vertical Bézier curve (BCHL) determine the curvature of the resulting surface: a longer handle results in a more convex surface (Fig. 4).

**Fig. 5:**
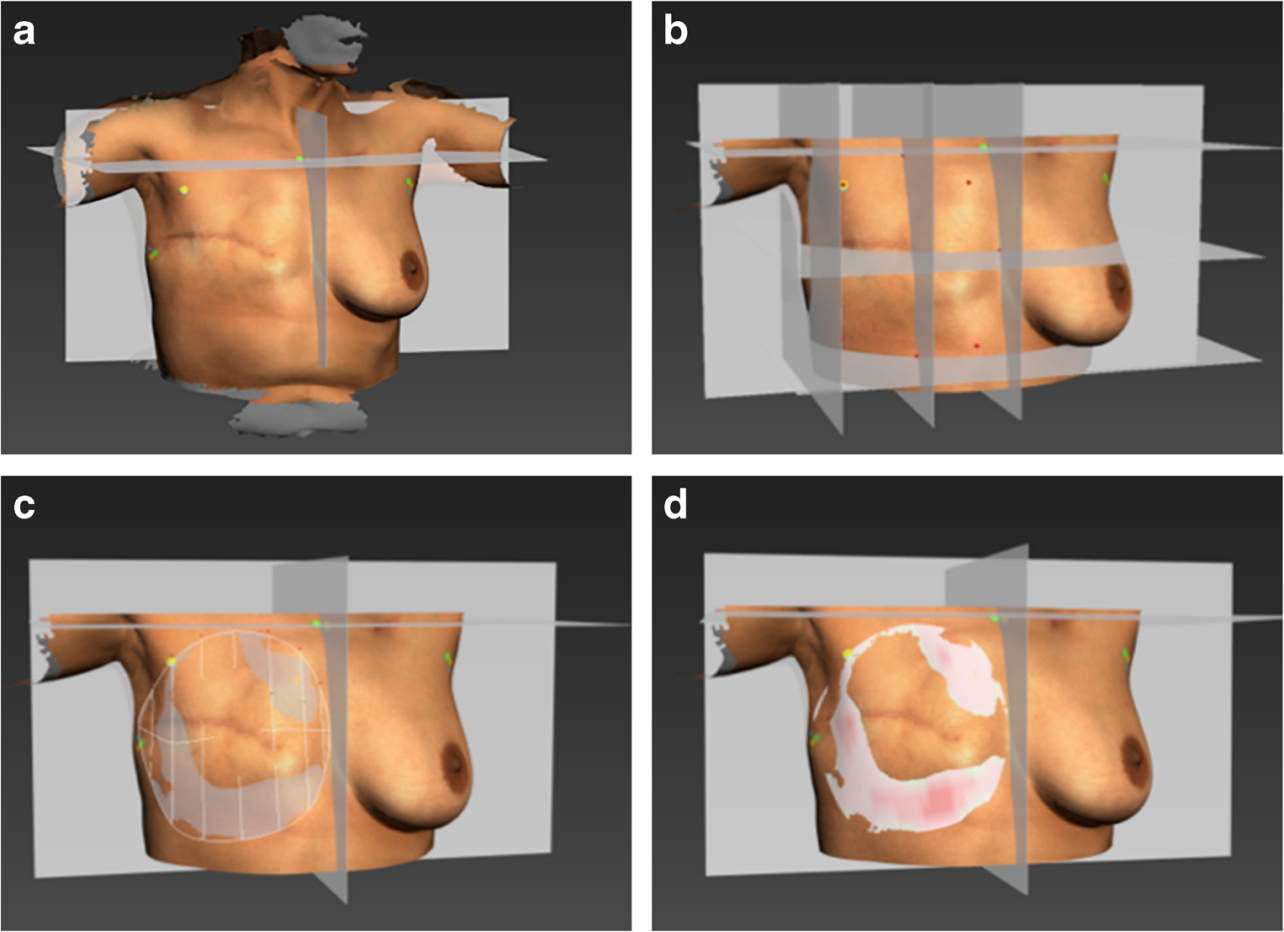
Method of simulating a chest wall. **a** The 3D surface image is imported and manually placed in the reference frame consisting of a sagittal, transverse, and frontal plane. The reference plane locations are based on the three breast boundary landmarks. **b** Eight breast boundary points (red dots) are placed on fixed locations according to the three breast boundary landmarks and reference planes. **c** A Bézier curve framework is created from to the eight breast boundary points and is curved according to the surrounding mesh curvature. Subsequently, the surface to simulate the chest wall is created within the Bézier curve frame. **d** A distance map is created on the simulated chest wall surface. The intensity of the red color correlates with the distance to the true chest wall. The green part of the surface is hidden behind the surface of the true chest wall

The steps of creating the simulated chest wall are as follows. The 3D surface images were placed in a three-plane reference frame which is based on the five placed landmarks (Fig. 5a). By using fixed relative distances in respect to the landmarks and the reference planes, eight breast boundary coordinates were selected on the 3D image (Fig. 5b). Subsequently, a smoothed curve was fitted through the eight coordinates to indicate the breast boundary, and vertical Bézier curves were created within the boundary (Fig. 5c). The direction of each Bézier curve handle was determined by the direction of the surface superior to the upper boundary spline vertices. The BCHL was a predetermined fraction of the Euclidian distance between the end points of each vertical Bézier curve. From the curve frame, a surface was created to simulate the chest wall (Fig. 5d).

For each of the 16 subjects, three different chest walls were simulated by using BCHLs with a factor 0.20, 0.33, and 0.50 of the distance between the end points of the Bézier curve (Fig. 4).

### Calculating the errors of the simulated chest walls

To determine the errors made by each of the 48 simulated chest walls, a distance map in respect to the true chest wall was created. To visualize the distances, each face was colored red or green depending on whether it was in front or behind the true chest wall, respectively (Fig. 5d). The intensity of the color correlates with the size of the measured distance. To calculate the volume error made by each simulated chest wall, the surface size of each simulated chest wall was multiplied by the mean relative distance to the true chest wall.

### Statistical analyses

All landmarks were grouped into three groups: the sternomanubrial-, the superolateral breast-, and midaxillary line landmarks. For each of the three groups, the means and standard deviations of intra- and interrater Euclidean distances were calculated. A paired difference *t* test was used to determine whether there was a difference between the intra- and interrater landmark distances.

For each of the three BCHL groups, the mean and standard deviations were calculated for the distance to the true chest wall and its resulting volume error. One sample *t* tests were performed to determine whether the means were different from zero.

A *p* value smaller than 0.05 was considered to be statistically significant. All statistical analyses were performed using SPSS version 22 (IBM Corporation, NY, USA).

## Results

Table 2 shows that the absolute intra- and interrater variability in marker placement was small (mean 3.5–6.7 mm). Furthermore, no statistically significant differences were found between the intra- and interrater variabilities of the sternomanubrial-, superolateral-, and midaxillary line markers (*p* = 0.08, *p* = 0.06, and *p* = 0.10, respectively). However, a trend towards the interrater variability being larger than the intrarater variability seems to be present.

**Table 2:** Intra- and interrater variability of the three landmark locations in 16 subjects

Landmark location	Intrarater distance Mean ± SD (mm)	Interrater distance Mean ± SD (mm)	*p* value*
Sternomanubrial joint (*n* = 16)	3.9 ± 2.6	5.8 ± 3.2	0.08
Superolateral breast (*n* = 32)	5.2 ± 2.5	6.7 ± 4.1	0.06
Midaxillary line (*n* = 32)	3.5 ± 2.2	4.6 ± 2.9	0.10

*p values were determined for the chances of the intra- and interrater distances being different

In Table 3, the mean and standard deviation of the distances between the simulated and true chest walls, the resulting volume errors of the simulated chest walls, and the mean surface area of the simulated chest walls are presented. The simulated chest walls created by a BCHLs of 0.33 showed the best approximation of a true chest wall (error of 4.6 ± 37 ml). The volume errors of the BCHLs 0.20 and 0.33 did not differ significantly from 0 (*p* = 0.21 and 0.63, respectively), while a BCHL of 0.50 did differ from 0 (*p* < 0.01).

**Table 3:** Mean surface area, mean distance error, and the resulting volume error of the simulated chest walls created with the three Bézier curve handle lengths (*n* = 16)

Bézier handle length	Surface area of the simulated chest wall Mean ± SD (cm^2^)	Distance error of the simulated chest wallMean ± SD (mm)	Resulting volume error Mean ± SD (ml)
0.20	283 ± 56	− 0.53 ± 1.4 (*p* = 0.15)	− 13 ± 40 (*p* = 0.21)
0.33	283 ± 53	0.13 ± 1.3 (*p* = 0.69)	4.6 ± 37 (*p* = 0.63)
0.50	289 ± 56	1.2 ± 1.3 (*p* < 0.01)	37 ± 41 (*p* < 0.01)

*p* values were determined for the chances of the error being 0

## Discussion

### Breast boundary landmarks

The newly introduced breast boundary landmarks in this study showed to be robust and can theoretically be used in patients with all body types. Furthermore, they can be used by multiple software applications. The requirement for a software application is that it either uses these specific landmarks to determine the breast boundary for a breast volume calculation or that it relies on a user-determined breast boundary without any preprogrammed landmarks. This means that, in addition to our own 3D BreAST software, the landmarks can be used with 3dMD Vultus software, which ships with every 3dMD camera setup that could be used to capture breasts.

For the lateral breast boundary, most authors use the lateral end of the inframammary fold [14–16, 19], while some use the posterior axillary line [17] or palpate the breast boundaries [10]. However, the lateral end of the inframammary fold is difficult to determine in adipose patients due to the fat roll that extends laterally from the breast to the back. The posterior axillary line can be determined in all patients, but it makes the breast boundary unnecessary large since the breast does not reach to the back. In our experience, palpating the lateral breast boundary in adipose patients is impossible. Our lateral landmark on the mid-axillary line can be used in adipose subjects, includes the axillary tail of the breast tissue, and shows no difference in intra- or interrater variability when placing it.

The superolateral breast boundary in published studies is determined by the lateral offshoot of the pectoral muscle [14], the common border of the upper arm and chest wall [17] or is arbitrarily chosen by the observer instead of being determined by anatomical landmarks [10, 19]. We chose the transition of the pectoral muscle into breast curvature as the superolateral boundary. This leaves room for interpretation, which explains the larger intra- and interrater variability.

The superior breast boundary is often defined at the level of the clavicle [14–17], by the sternal notch height [19], by palpation [10], or the location where the skin wrinkles when the subject lifts her breast [13]. Defining the superior breast boundary near the clavicle or the sternal notch introduces an undesirable large breast boundary, because superiorly the breast tissue ends lower at the second rib. In our experience, palpating the superior breast boundary is mostly infeasible, especially when the patient is adipose. Lifting the breast to cause a wrinkle is an interesting method to define the upper boundary; however, in adipose subjects or subjects with small breasts, this is difficult to perform. Our proposed superior boundary landmark at the sternomanubrial joint is very well palpable in slim and adipose patient and does not introduce an unnecessary large breast boundary, because the second rib is the anatomical upper limit of the breast.

Only Henseler et al. determined the intrarater variability for digitally placed breast boundary landmarks [18]. Because the authors did not use a landmark for the superior and superolateral breast boundary, only their lateral breast boundary landmark is comparable to our set. For this landmark, they reported an average intrarater distance of 4.5 ± 5.2 mm, which is larger than the 3.5 ± 2.2 mm we found for our lateral marker. This puts our results in perspective and suggests that our marker is more robust. No interrater variability or correlations between the marker set and the breast volume measurements were determined in their research.

### Chest wall simulation

The three tested chest wall simulation types with Bézier curve handle lengths of a factor 0.20, 0.33, and 0.50 of the distance between the end points of the curve yielded an average mean volume error of -13 ± 40, 4.6 ± 37, and 37 ± 41 ml, respectively. Together with our experience, the trend in these results suggests that the Bézier curve handle length of 0.33 is the best choice to use in our algorithm.

To our knowledge, only Kovacs et al. [14] tried to determine the accuracy of their simulated chest wall to validate their breast volume calculating software. They calculated the mean distance between 10 simulated chest walls from 3D surface images to segmented chest walls from a corresponding MRI scan. The mean error and standard deviation from their error measurements was 1.80 ± 3.77 mm. When using the mean surface area of a simulated chest wall from our study (285 cm^2^), this would result in an average volume error of 51.3 ± 107 ml. This is a factor 10 higher than a chest wall simulated with the most accurate parameter from our study. Furthermore, the approach introduces errors from the segmentation of the MRI scan and the alignment of the 3D surface image with the MRI. It was not described how the chest wall deviation was calculated in their study.

Two studies validated their algorithm with actual breast volumes after resection, instead of a chest wall [10, 12]. Yip et al. and Losken et al. reported a mean error of 46.7 ± 180 and  -14.4 ± 61.8 ml, respectively. Both these means and standard deviations are larger than the volume errors found in our study.

Despite the exclusion of subjects with severe scars, deformations of the chest walls were present in most subjects. Furthermore, the contractions caused by the scarring sometimes caused sharp corners at the pectoral muscle, which the simulated chest wall did not follow accurately. Therefore, the volume errors made by the simulated chest walls in this study are probably slightly overestimated.

### Calculating a breast volume from a 3D photo in clinical practice

**Fig. 6:**
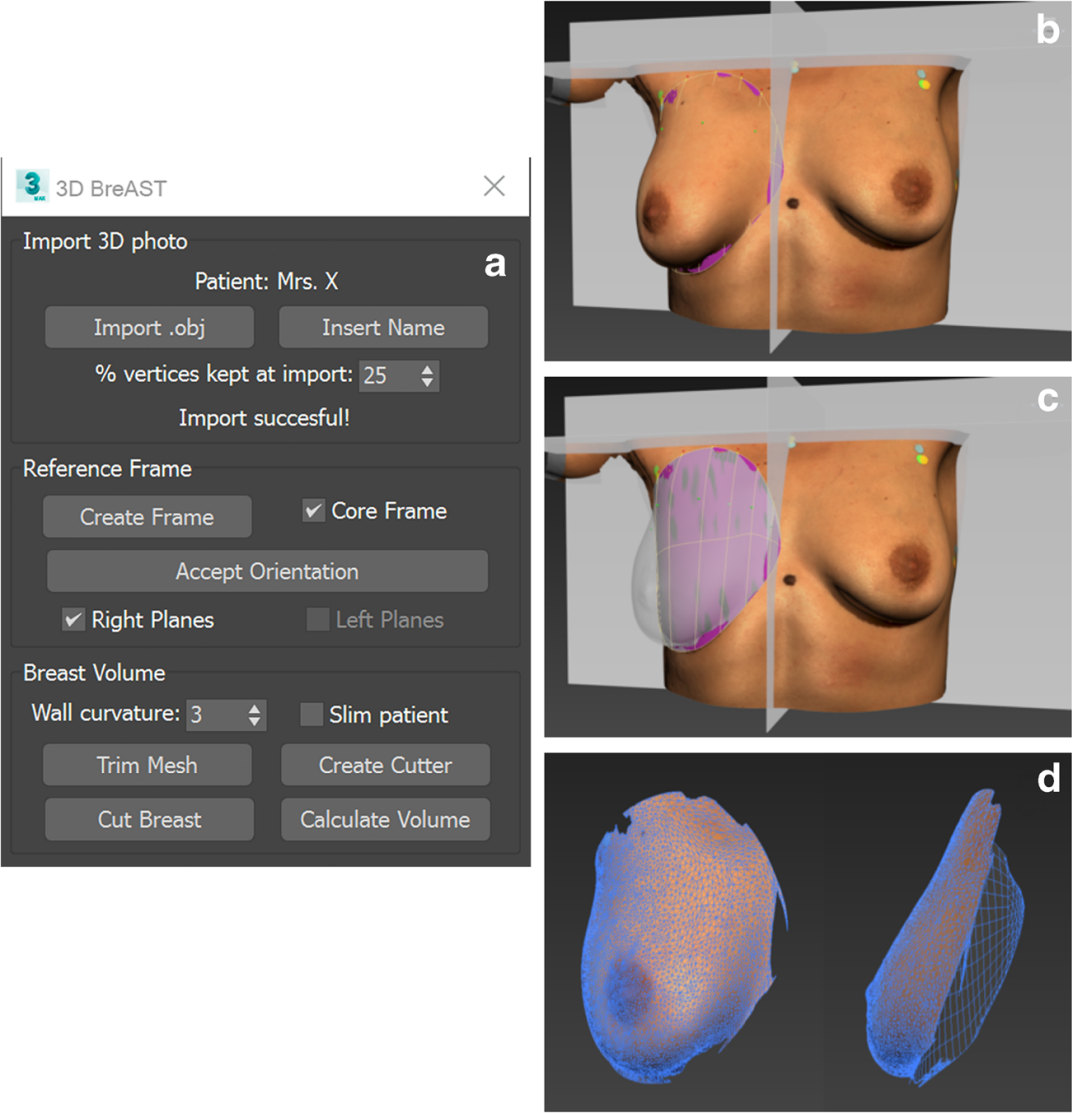
User interface and steps to use the 3D BreAST. **a** The user interface. **b** The 3D photo is placed in the reference frame and the landmarks that indicate the breast boundary are selected by the user. **c** The simulated chest wall that was created from the selected breast boundary is shown by making the breast surface transparent. **d** The cut breast which was capped at the back by the simulated chest wall

To calculate a breast volume from a 3D photo with the 3D BreAST software, one has to perform the following steps. (1) After placing stickers on the sternomanubrial-, superolateral breast-, and midaxillary anatomical landmarks, capture a 3D photo while the subject spreads her arms and exhales normally. (2) Open the 3D BreAST software (Fig. 6a) and import the 3D photo by clicking the “Import .obj” button. (3) Create a reference frame by clicking the “Create Frame” button. (4) Position the 3D photo correctly and click “Accept Orientation”. (6) Click “Create Cutter” and select the breast boundary by clicking on the landmarks to simulate a chest wall behind the breast surface (Fig. 6b). (7) Cut the breast from the 3D photo with the simulated chest wall by clicking “Cut Breast” (Fig 6c, d). (8) Click “Calculate Volume” to print the breast volume on the screen.

## Conclusion

This study shows that we have identified a robust set of breast boundary landmarks and developed an accurate chest wall simulation algorithm for breast volume calculations from 3D surface images. These landmarks can potentially be used by any software application that incorporates these landmarks to define the breast boundary. This validation study is an important step in introducing our 3D BreAST software in clinical practice to routinely calculate breast volumes for surgical planning. The next step is to compare the accuracy of our method with existing methods.

## References

[1] Hudson D (2004). Factors determining shape and symmetry in immediate breast reconstruction. Ann Plast Surg.

[2] Tezel E, Numanoğlu A (2000). Practical do-it-yourself device for accurate volume measurement of breast. Plast Reconstr Surg.

[3] Wilkie T, Ship AG (1976). Volumetric breast measurement during surgery. Aesthetic Plast Surg.

[4] Tegtmeier R (1978). Volumetric breast measurement during surgery. Ann Plast Surg.

[5] Campaigne BNN, Katch VLL, Freedson P, Sady S, Katch FI (1979). Measurement of breast volume in females: description of a reliable method. Ann Hum Biol.

[6] Edsander-Nord A, Wickman M, Jurell G (1996). Measurement of breast volume with thermoplastic casts. Scand J Plast Reconstr Surg Hand Surg.

[7] Grossman AJ, Roudner LA (1980). A simple means for accurate breast volume determination. Plast Reconstr Surg.

[8] Kalbhen CL, McGill JJ, Fendley PM, Corrigan KW, Angelats J (1999). Mammographic determination of breast volume: comparing different methods. Am J Roentgenol.

[9] Caruso MK, Guillot TS, Nguyen T, Greenway FL (2006). The cost effectiveness of three different measures of breast volume. Aesthetic Plast Surg.

[10] Yip JM, Mouratova N, Jeffery RM, Veitch DE, Woodman RJ, Dean NR (2012). Accurate assessment of breast volume. Ann Plast Surg.

[11] Mailey B, Freel A, Wong R, Pointer DT, Khoobehi K (2013). Clinical accuracy and reproducibility of portrait 3D surgical simulation platform in breast augmentation. Aesthet Surg J.

[12] Losken A, Seify H, Denson DD, Paredes AA, Carlson GW (2005). Validating three-dimensional imaging of the breast. Ann Plast Surg.

[13] Hoeffelin H, Jacquemin D, Defaweux V, Nizet JL (2014). A methodological evaluation of volumetric measurement techniques including three-dimensional imaging in breast surgery. Biomed Res Int.

[14] Kovacs L, Eder M, Hollweck R, Zimmermann A, Settles M, Schneider A, Udosic K, Schwenzer-Zimmerer K, Papadopulos NA, Biemer E (2006). New aspects of breast volume measurement using 3-dimensional surface imaging. Ann Plast Surg.

[15] Eder M, Waldenfels FV, Swobodnik A, Klöppel M, Pape A-K, Schuster T (2012). Objective breast symmetry evaluation using 3-D surface imaging. Breast.

[16] Eder M, Grabhorn A, Waldenfels FV, Schuster T, Papadopulos NA, Machens H-G (2013). Prediction of breast resection weight in reduction Mammaplasty based on 3-dimensional surface imaging. Surg Innov.

[17] Liu C, Luan J, Ji K, Sun J (2012). Measuring volumetric change after augmentation mammaplasty using a three-dimensional scanning technique: an innovative method. Aesthetic Plast Surg.

[18] Henseler H, Smith J, Bowman A, Khambay BS, Ju X, Ayoub A (2012). Investigation into variation and errors of a three-dimensional breast imaging system using multiple stereo cameras. J Plast Reconstr Aesthet Surg.

[19] Mioton LM, Jordan SW, Kim JYS (2015). A prospective analysis of dynamic loss of breast projection in tissue expander-implant reconstruction. Arch Plast Surg.

[20] Reece GP, Merchant F, Andon J, Khatam H, Ravi-Chandar K, Weston J, Fingeret MC, Lane C, Duncan K, Markey MK (2015). 3D surface imaging of the human female torso in upright to supine positions. Med Eng Phys.

[21] Georgii J, Eder M, Burger K, Klotz S, Ferstl F, Kovacs L, Westermann R (2014). A computational tool for preoperative breast augmentation planning in aesthetic plastic surgery. IEEE J Biomed Health Informat.

[22] Tomita K, Yano K, Hata Y, Nishibayashi A, Hosokawa K (2015). DIEP flap breast reconstruction using 3-dimensional surface imaging and a printed mold. Plast Reconstr Surg Glob Open.

[23] Eriksen C, Lindgren EN, Olivecrona H, Frisell J, Stark B (2011). Evaluation of volume and shape of breasts: comparison between traditional and three-dimensional techniques. J Plast Surg Hand Surg.

